# CD8^+^ t cell-mediated regulation of synaptic integrity in Alzheimer’s disease

**DOI:** 10.3389/fimmu.2026.1903001

**Published:** 2026-10-01

**Authors:** Hua Fu, Jing Yang, Miao Zhang

**Affiliations:** 1 The Second Clinical Medical College, Heilongjiang University of Chinese Medicine, Harbin, Heilongjiang, China; 2 The Second Affiliated Hospital of Heilongjiang University of Chinese Medicine, Harbin, Heilongjiang, China

**Keywords:** Alzheimer’s disease, CD8+ T cells, complement cascade, granzyme K, MHC class I, microglia, neuroimmunology, synaptic integrity

## Abstract

Alzheimer’s disease (AD) has traditionally been interpreted through amyloid, tau, and innate immune frameworks, with the complement cascade long regarded as the principal driver of aberrant synaptic pruning. Synaptic loss is increasingly recognized as one of the strongest correlates of cognitive decline. This review examines an emerging CD8^+^ T cell–synapse axis that extends beyond complement-centric models. We discuss how brain-infiltrating CD8^+^ T cells intersect with synaptic integrity through MHC-I-dependent and MHC-I-independent mechanisms, including IFN-γ–mediated microglial reprogramming and granzyme K–driven non-canonical complement activation. Early effector-like subsets may promote neuroinflammation and synaptic injury, whereas later tissue-resident or regulatory populations can exert protective functions by gating microglial states. The net outcome depends on disease stage, antigenic milieu, anatomical niche, and microglial context. We emphasize that direct antigen-specific, MHC-I-restricted synaptic attack remains unproven in human AD, and that observed T-cell infiltration may reflect reverse causality. Translational limitations across species are evaluated, and we outline subset-specific immunomodulation, ApoE4-stratified strategies, and biomarker-guided trial designs as future directions. Overall, CD8^+^ T cells are positioned not as uniform cytotoxic executors, but as dynamic, context-dependent regulators of synaptic outcomes in AD.

## 1 Introduction

Alzheimer’s disease (AD) is the most prevalent cause of dementia. It affects over 55 million individuals worldwide, and its prevalence is projected to nearly triple by 2050 (1). The global economic burden of dementia exceeds $1 trillion annually (2). Although amyloid-β plaques and neurofibrillary tangles remain the defining pathological hallmarks of AD, synaptic degeneration is increasingly recognized as a fundamental pathological basis of cognitive dysfunction (3). Cerebrospinal fluid (CSF) synaptic biomarkers predict longitudinal cognitive impairment beyond traditional amyloid and tau metrics, and a CSF synaptic protein ratio (YWHAG: NPTX2) begins to rise approximately two decades before estimated symptom onset in autosomal dominant AD (4). This gap suggests that the mechanisms driving synaptic elimination remain incompletely understood.

The neuroinflammatory framework of AD has long been anchored to innate immunity. The complement cascade is championed as the principal mechanism driving aberrant synaptic pruning (5). C1q deposition tags synapses for microglial engulfment. Genetic deletion of classical pathway components rescues synapse loss in transgenic models (6). Nevertheless, region- and age-dependent synaptic loss occurs in vulnerable circuits despite robust perisynaptic complement tagging. Complement receptor signaling does not fully explain the spatial and temporal mismatches between synapse vulnerability and complement activation (7, 8). These limitations point toward alternative immune-mediated pruning pathways that operate in parallel with C1q-C3 signaling.

Recent evidence suggests that brain-infiltrating CD8^+^ cytotoxic T lymphocytes may play a role in AD pathophysiology. Human studies have identified clonally expanded, antigen-experienced CD8^+^ T cells in AD CSF and CD3^+^ T-cell infiltrates in brain parenchyma with their distributions correlating more robustly with tau burden than with amyloid deposition (9, 10). Rodent models demonstrate that these cells infiltrate amyloid-laden brain regions and alter synapse-related gene expression (11, 12). Importantly, CD8^+^ T cells exert context- and disease stage-dependent effects. Some subsets promote neuroinflammation and neurodegeneration. Others exert regulatory or neuroprotective effects that limit pathology (13, 14). Nevertheless, this framework remains hypothetical to a significant extent. Notably, direct evidence for antigen-specific, MHC-I-restricted CD8^+^ T-cell recognition of neurons or synapses in the human AD brain is lacking. Neuronal MHC-I expression in the adult human cortex remains largely uncharacterized under physiological conditions, and no cognate neuronal or synaptic antigen has been identified for clonally expanded CD8^+^ T cells in AD tissue (9, 15). Consequently, the MHC-I–synapse axis, while mechanistically plausible, remains extrapolated from rodent models and non-AD viral paradigms rather than validated in human disease. Furthermore, the observed association between CD8^+^ T cells infiltration and synaptic pathology may reflect reverse causality. AD pathology itself may recruit and activate CD8^+^ T cells as a secondary response through chemokine release, blood-brain barrier disruption, and antigen exposure. This would mean that these cells do not initiate synaptic damage (16, 17). Recent reviews have examined the involvement of adaptive immunity, including CD8^+^ T cells, in AD neuroinflammation (18–20). However, these prior syntheses have primarily situated T cell biology within broad neuroinflammatory or neurodegenerative frameworks. The present review specifically examines how brain-infiltrating CD8^+^ T cell programs intersect with synaptic integrity in AD. It integrates pathogenic and protective functional states, evaluating translational constraints across species, and identifying unresolved mechanistic questions that limit causal inference and future clinical translation. This review is presented as a narrative synthesis. A literature search was conducted in PubMed, Web of Science, and Google Scholar from inception to August 3, 2026, using keywords related to CD8+ T cells, Alzheimer’s disease, synaptic integrity, complement cascade, microglia, MHC class I, and granzyme K. Articles were selected thematically to highlight mechanistic and translational advances, with representative rather than exhaustive coverage.

## 2 Immune pathways governing synaptic integrity in AD: complement and the MHC-I axis

### 2.1 The complement cascade in synaptic homeostasis and Alzheimer’s pathology

The complement cascade is one of the most extensively studied innate immune pathways that regulate synaptic elimination in the central nervous system (CNS) (21). Under physiological conditions, complement proteins are locally produced by microglia, astrocytes, and neurons. They participate in immune surveillance, debris clearance, and glial communication (22).

Beyond debris removal, the complement system also mediates the refinement of neural circuits during development. In this context, C1q triggers the classical complement cascade, which cleaves C3 into C3b and its degradation product iC3b. These molecules act as opsonins that tag synapses for elimination. Microglia expressing complement receptor 3 (CR3; CD11b/CD18) then recognize and engulf these tagged synapses (23, 24). This mechanism was first characterized in the developing visual system, where C1q and C3 mediate the elimination of immature retinogeniculate synapses to refine eye-specific segregation (24). Subsequent studies extended this framework to postnatal thalamic wiring. They demonstrated that microglia shape neural connectivity in an activity-dependent and complement-dependent manner (23). Collectively, these findings establish a key principle. Complement-mediated pruning is not a non-selective or ubiquitous process. Instead, it is a finely tuned mechanism constrained by neural activity and synapse specificity.

The pathological redeployment of complement-dependent synapse elimination was first demonstrated in mouse models of glaucoma. In these models, C1q is upregulated and relocalized to retinal ganglion cell synapses at early disease stages. This occurs before axonal degeneration and neuronal death, suggesting that developmental pruning modules can be mistakenly re-engaged in neurodegenerative conditions (25). These findings in glaucoma established a precedent for complement-mediated early synaptic injury that has since been extended to AD models. Similar findings have been reported in AD transgenic mice. C1q levels are elevated and deposited onto hippocampal synapses before overt plaque formation. Blocking C1q activation or deleting CR3 attenuates early synapse loss. In addition, antibody-mediated C1q inhibition rescues hippocampal long-term potentiation impaired by soluble amyloid-β (Aβ) oligomers (6). In aged APP/PS1 mice, C3 deficiency protects against neurodegeneration and preserves synaptic integrity, even when amyloid burden is increased. This suggests that complement-mediated synaptic toxicity can be dissociated from plaque clearance, at least in part (26). In tauopathy models, C1q accumulates at postsynaptic densities and correlates with phospho-tau burden. C1q-blocking antibodies rescue synapse density in Tau-P301S mice. These observations position the complement cascade as a potential convergence point for both Aβ-driven and tau-driven synaptopathy (27).

Recent studies have revealed that complement-mediated synaptic damage involves greater cellular complexity beyond the classical model of extracellular opsonization and engulfment. Scott-Hewitt and colleagues found that microglia-derived C1q is internalized by neurons through endocytosis and subsequently integrates into ribonucleoprotein complexes via RNA-dependent liquid-liquid phase separation. This process impairs neuronal protein synthesis in an age-dependent manner (28). This finding suggests that complement components may compromise synaptic function not only by tagging synapses extracellularly and promoting engulfment, but also by disrupting intracellular proteostasis. Meanwhile, Dejanovic and colleagues uncovered a functional division of labor between astrocytes and microglia in complement-dependent synapse elimination. Astrocytic lysosomes were enriched in excitatory synapse marker proteins, whereas microglial lysosomes contained more inhibitory synapse material. In TauP301S mice, C1q deletion reduced synapse engulfment in both astrocytes and microglia. Furthermore, in TauPS2APP; TREM2 knockout mice with impaired microglial phagocytosis, astrocytes compensated by engulfing more inhibitory synapses around plaques (29). This suggests that dynamic functional compensation and task allocation exist among different glial populations during complement-associated synaptic elimination.

It is important to emphasize that the complement system is not intrinsically pathogenic. Its functions in the CNS are highly context-dependent and exhibit duality. Under physiological conditions, C1q, independent of the classical cascade, enhances microglial clearance of apoptotic neurons and neuronal blebs while concomitantly suppressing proinflammatory cytokine production, including IL-1α, IL-1β, TNF-α, and IL-6 (30). This immunomodulatory action preserves the immune-quiescent microenvironment of the healthy brain. The anaphylatoxins C3a and C5a, generated during complement activation, act through their respective receptors C3a receptor (C3aR) and C5a receptor 1 (C5aR1), which are expressed on astrocytes, microglia, and neurons. These receptor–ligand interactions contribute to chemotaxis, inflammatory modulation, the maintenance of blood–brain barrier integrity, and the regulation of adaptive immune responses within the CNS (21, 22, 31). Complement components also provide direct support to neurons. At the molecular level, C1q directly upregulates neuronal genes associated with cytoskeleton function, cholesterol metabolism, and neurotrophic signaling, thereby promoting neuronal survival and neurite outgrowth (32). C1q further protects neurons against amyloid-β neurotoxicity by inducing the neuronal receptors LDL receptor-related protein 1B and G protein-coupled receptor 6 (33). Similarly, C3a and C5a exert context-dependent effects on neuronal function. C3a modulates neural progenitor cell differentiation and migration (34), and the C3/C3aR axis exerts dissociable effects on the survival and morphological maturation of adult-born hippocampal neurons (35). C5a, acting through neuronal C5aR1, protects against glutamate-mediated excitotoxicity by regulating expression of the glutamate receptor subunit 2 (31, 36). These observations indicate that complement signaling is not merely a downstream effector of immune activation but an active participant in glial-neuronal communication and synaptic plasticity. This duality is likewise evident in disease settings. In AD models, C5aR1 signaling promotes region-dependent and age-dependent synaptic pruning, yet selective C5aR1 antagonism preserves cognitive function and limits detrimental glial polarization while largely sparing beneficial complement functions (7). In the adult brain, basal complement activity thus contributes to ongoing immune surveillance and the maintenance of synaptic homeostasis, whereas its dysregulation in AD reflects a quantitative and contextual imbalance rather than the engagement of an intrinsically destructive pathway. This view is further supported by multiple endogenous inhibitory mechanisms that collectively constrain complement-mediated excessive synaptic elimination. TREM2 protects against complement-mediated synaptic loss by directly binding C1q and suppressing classical pathway activation. In human AD brains, TREM2-C1q complex formation is associated with lower C3 deposition and higher synaptic protein levels, and TREM2 haploinsufficiency increases complement-mediated microglial engulfment of synapses in tauopathy mice (37). Similarly, the neuronal pentraxin 2 binds C1q and blocks classical complement pathway activation, and Nptx2 deletion leads to C1q-dependent synapse elimination through microglial pruning in the adult brain (38). These lines of evidence indicate that complement-mediated synaptic pruning is subject to active and finely tuned negative regulation under physiological conditions, and that the aberrant synapse loss observed in AD may partly arise from the failure of such endogenous homeostatic mechanisms.

Despite the mechanistic robustness of complement-mediated synaptic pruning in experimental models, the complement-centric model of AD synaptopathy faces several challenges. These challenges relate to temporal precision, spatial specificity, and cross-species generalizability. First, at the temporal level, synaptic density reduction in early-stage AD, as measured by synaptic vesicle glycoprotein 2A (SV2A) PET, correlates with cognitive performance across multiple neocortical regions (39). Because such imaging measures do not resolve the underlying molecular drivers, current human evidence cannot establish whether complement activation precedes or follows synaptic loss. The temporal ordering required by a causal complement-centric model thus remains untested in patients and complement activation may not serve as the primary trigger driving synapse loss in all circumstances. At certain disease stages or within specific pathological contexts, it may function as a secondary amplifying mechanism that responds to pre-existing synaptic stress and network imbalance. Second, at the spatial level, the most vulnerable regions in AD are hippocampal CA1 and entorhinal cortex layer II. These regions differ from the developing visual and thalamic circuits in which complement-dependent pruning was originally defined (23, 24), and whether comparable regional rules operate in the adult human brain remains unresolved. Connectome-based analyses demonstrate that the spatial pattern of tau accumulation in AD is associated with functional network architecture, supporting transneuronal tau propagation. This suggests that local complement-mediated pruning may be only one of multiple determinants of regional vulnerability (40). Third, at the species level, multiple AD mouse models show that genetic or pharmacological inhibition of complement components rescues synapse density and ameliorates pathological phenotypes. However, longitudinal human studies have revealed a seemingly paradoxical finding: higher baseline CSF C3 and factor H levels are associated with slower cognitive decline in individuals with mild cognitive impairment (41). As with the associations discussed for CD8^+^ T-cell infiltration, this correlation is compatible with a homeostatic role for complement in the human brain, yet it cannot establish temporal precedence or directionality. It remains equally plausible that more aggressive synaptic or inflammatory pathology in rapid decliners drives complement consumption, yielding lower measurable levels, rather than indicating that high baseline complement is causally protective. Consequently, this observation remains subject to the same reverse-causality caution applied elsewhere in this review.

In summary, the complement pathway is undoubtedly an important component of synaptic immunopathology in AD. However, its role is likely stage-dependent, region-dependent, and species-dependent. It is therefore insufficient on its own to fully account for the entire spatiotemporal topography of synaptic degeneration in AD.

### 2.2 MHC-I at the interface of adaptive immunity and synaptic biology

The regulation of synaptic integrity in AD is often discussed primarily within the framework of innate immunity, particularly complement-dependent synaptic pruning (6, 24). However, accumulating evidence suggests that adaptive immune mechanisms may also contribute to synaptic vulnerability in the CNS (9, 13). At the center of this interface is major histocompatibility complex class I (MHC-I), which links endogenous antigen presentation to CD8^+^ cytotoxic T-cell recognition while also participating in neuronal structural and synaptic regulation (42, 43). This dual role positions MHC-I as a potential molecular bridge between adaptive immunity and synaptic biology (44).

Under physiological conditions, neuronal MHC-I expression in the adult brain is generally low and tightly regulated. Studies in rodents have shown that MHC-I transcripts can be detected in multiple brain regions including the hippocampus, cortex, and lateral geniculate nucleus during development, and are developmentally downregulated as neonatal mice reach adulthood (45), although MHC-I protein remains detectable at both pre- and postsynaptic sites in the adult visual cortex (46). In aged rat motoneurons, mRNA for MHC-I and β2m increases with age (47). In contrast, neuronal MHC-I protein expression in the healthy adult human cortex and hippocampus has not been reported, whereas catecholaminergic neurons in the substantia nigra and locus coeruleus do express MHC-I under normal conditions (15). Nonetheless, recent work indicates that neuronal MHC-I is not entirely absent but instead exhibits cell type–specific and context-dependent expression, with particular relevance to aging-related neuronal vulnerability and ApoE-associated regulation (48). These observations suggest that neuronal MHC-I provides a plausible, though tightly constrained, substrate through which adaptive immune recognition could intersect with synaptic homeostasis.

In inflammatory settings, this constraint may be partially relaxed. Experimental studies have shown that neuronal MHC-I can be induced by inflammatory signals such as interferon-γ (IFN-γ), raising the possibility that stressed neurons may acquire limited antigen-presenting capacity (49, 50). In AD, this possibility is supported indirectly by evidence that disease-associated stressors can perturb MHC-I-related signaling (51, 52), and that neuronal apolipoprotein E (ApoE) may promote MHC-I upregulation under neurodegenerative conditions (48). Although this mechanism is supported in several experimental systems, direct evidence that antigen-specific CD8^+^ T-cell recognition of neurons occurs in human AD brain and contributes to synaptic loss remains limited. At present, neuronal antigen presentation in AD is best regarded as a mechanistically plausible but incompletely validated pathway.

Importantly, adaptive immune regulation in the AD brain is unlikely to depend solely on neurons themselves. Microglia constitute the principal MHC-I-expressing antigen-presenting population within the CNS, and their MHC-I expression is upregulated with aging and AD progression in both mouse models and human tissue (53). Thus, the influence of adaptive immunity on synaptic integrity may proceed through at least two non-mutually exclusive routes: a direct route, in which stressed neurons acquire limited immune visibility, and an indirect route, in which microglia mediate antigen presentation, inflammatory amplification, and downstream effects on synaptic homeostasis (12, 44). Under current evidence, the latter pathway is likely to be more broadly relevant.

In addition, MHC-I may influence synaptic function through mechanisms not strictly dependent on antigen presentation or T-cell recognition. Endogenous MHC-I tonically inhibits NMDAR function and controls downstream NMDAR-induced AMPA receptor trafficking during the expression of plasticity. MHC-I deficiency enhances NMDAR-mediated currents and alters AMPA/NMDA ratios at hippocampal synapses without changing total receptor levels (43). Furthermore, the cytoplasmic tail of MHC-I proteins regulates spine and synapse density and morphology in hippocampal neurons through outside-in signaling, modulating glutamate receptor expression independently of antigen presentation and T-cell receptor (TCR) recognition (54, 55). The β2-microglobulin component of MHC-I can also function as an endogenous NMDAR antagonist, impairing synaptic function when elevated in the circulation (56). These non-immune functions further broaden the potential scope of adaptive immune involvement in AD synaptopathy.

Taken together, these considerations point toward a broader conceptual framework within which synaptic regulation in AD may be shaped not only by complement-driven innate immune programs but also by an emerging T cell–synapse axis. However, whether CD8^+^ T-cell accumulation represents a primary driver of synaptic pathology, a secondary amplifier of ongoing neurodegeneration, or a reactive consequence of tissue injury remains unresolved.

## 3 CD8^+^ T cells and synaptic integrity in AD

### 3.1 Functional overview and disease-stage dynamics of CD8^+^ T cells

CD8^+^ cytotoxic T lymphocytes represent a specialized lineage of adaptive immune cells that undergo thymic selection and peripheral maturation to acquire the capacity for antigen-specific surveillance and targeted cellular elimination. Following antigen encounter, naive CD8^+^ T cells undergo extensive clonal expansion and differentiate into effector populations capable of executing antimicrobial and antitumor functions (57). Their principal cytotoxic mechanism involves the directed exocytosis of lytic granules containing perforin and granzymes, which induce target-cell apoptosis, together with the secretion of proinflammatory cytokines such as IFN-γ and TNF-α that amplify local immune responses (58). These effector functions are tightly regulated by TCR recognition of peptide antigens presented by MHC class I molecules, thereby ensuring antigen specificity while limiting unintended tissue damage (59).

Beyond circulating effector and central memory populations, tissue-resident memory T cells constitute a non-recirculating subset that establishes long-term residence within peripheral tissues, including mucosal surfaces, barrier sites, and the CNS. These cells provide rapid frontline defense against previously encountered pathogens through local cytokine production and cytotoxicity, independently of replenishment from the bloodstream (60). In the healthy CNS, T cells patrol the meninges and perivascular spaces under steady-state conditions, contributing to immune surveillance without necessarily compromising neural function (61). Thus, the presence of T cells in these compartments reflects a basal level of immunological vigilance and does not inherently imply tissue destruction. However, tissue-resident memory CD8^+^ T cells can also cooperate with other adaptive immune cells to drive compartmentalized immunopathology in the CNS, underscoring the context-dependent nature of their functional output (62).

The functional impact of CD8^+^ T cells extends beyond beneficial pathogen clearance and tumor surveillance to include potentially harmful consequences when immune responses become dysregulated or excessively prolonged. In neuronal contexts, CD8^+^ T cells can mediate perforin-dependent electrical silencing of target cells, a rapid effect that precedes neuronal cell death yet is not necessarily causally linked to it. This illustrates how molecular machinery normally used to eliminate infected cells can transiently disrupt electrically excitable tissues (63). Chronic antigen stimulation can drive T cells toward exhaustion, characterized by diminished effector function and altered transcriptional programs, or alternatively toward sustained activation states that perpetuate host-tissue damage (64, 65). In parallel, the tight spatial and temporal regulation of MHC-I expression in hippocampal neurons suggests that inappropriate MHC-I dysregulation may disrupt synaptic remodeling and neuronal structural integrity (45). Together, these observations highlight the dual nature of CD8^+^ T-cell function: these cells can act as essential guardians of immune surveillance, but they can also become mediators of neural dysfunction when regulatory mechanisms fail.

The impact of CD8^+^ T cells on synaptic integrity in AD is unlikely to be static. Instead, available evidence suggests that their functions evolve across disease progression. Recent studies in AD mouse models demonstrate a stage-dependent transition. Early brain-infiltrating, clonally expanded CD8^+^ T cells exhibit effector-like and pathogenic programs. Later populations, by contrast, acquire tissue-resident or exhaustion-like features with corresponding functional shifts (13). In human AD, peripheral CD8^+^ T effector memory subsets accumulate during the prodromal mild cognitive impairment stage and display phenotypic alterations linked to CSF biomarkers and cognitive decline (66). CSF immune profiling has revealed progressive immune dysregulation during healthy brain aging and cognitive impairment, with T-cell clonality and inflammatory signatures increasing along the cognitive decline trajectory (67). These findings are compatible with a contributory role of CD8^+^ T cells, but they are equally consistent with recruitment secondary to pre-existing synaptic injury, complement activation, or neurodegeneration. Although human data are directionally consistent with temporal heterogeneity, they remain largely cross-sectional and regionally variable. Accordingly, the relationship between CD8^+^ T-cell abundance and amyloid or tau pathology should not be interpreted as linear or uniform across disease stages. Notably, chronic stress has been shown to regulate AD progression via selective enrichment of CD8^+^ T cells, suggesting that systemic physiological states can modulate the adaptive immune contribution to neurodegeneration (68).

This temporal plasticity may help reconcile otherwise divergent observations. Early-stage disease may favor CD8^+^ T-cell states associated with immune surveillance, inflammatory amplification, and local neuronal stress, whereas later-stage lesions may preferentially harbor functionally remodeled populations with reduced cytotoxic potential and greater tissue adaptation. Checkpoint-associated pathways, including PD-1/PD-L1 and TIM-3-related signaling, may contribute to this functional remodeling within the chronically inflamed brain microenvironment. In human AD, PD-L1 is elevated in CSF and brain tissue (69), and TIM-3/HAVCR2 regulates microglial responses in mice (70). However, direct evidence for bona fide CD8^+^ T-cell exhaustion in human AD remains limited. Without stage-stratified sampling, these temporally distinct phenotypes are difficult to resolve in post-mortem tissue. This makes it challenging to determine whether CD8^+^ T cells actively contribute to synaptic injury at defined disease stages or instead accumulate as part of a later inflammatory response to ongoing neurodegeneration (71).

### 3.2 Pathogenic CD8^+^ T-cell programs and synaptic damage

Consistent with this framework of functional heterogeneity, the pathogenic programs of CD8^+^ T cells in AD have been characterized across human observational studies and experimental models. Gate et al. (2020) identified clonally expanded CD8^+^ effector memory CD45RA^+^ cells in the CSF of AD patients, with peripheral CD8^+^ effector memory CD45RA^+^ cells negatively associated with cognitive performance (9). Post-mortem immunohistochemical studies have documented extravascular CD3^+^ T-cell infiltrates in the AD brain parenchyma, with their spatial distribution correlating more robustly with tau burden than with amyloid deposition (10). CD8^+^ T cells are frequently observed in parenchymal niches vulnerable to neurodegeneration (10). These findings are compatible with a contributory role of CD8^+^ T cells, but they are equally consistent with recruitment secondary to pre-existing synaptic injury, complement activation, or neurodegeneration. Consequently, human observational data alone cannot establish whether CD8^+^ T-cell infiltration initiates synaptic damage, amplifies ongoing degeneration, or represents an epiphenomenal immune response to neuronal stress. At the functional level, brain-infiltrating CD8^+^ T cells in AD models exhibit effector-like and proinflammatory programs. In P301S tauopathy mice, IFN-γ-producing T cells establish functional crosstalk with microglia, which acquire an antigen-presenting phenotype and amplify neuroinflammatory injury (51). Similarly, hippocampal T-cell infiltration promotes neuroinflammation and cognitive decline in tau transgenic mice, and systemic CD3 blockade reverses spatial memory impairment (72). In a 3D human neuroimmune axis model, infiltrating CD8^+^ T cells exacerbate AD pathology primarily by synergizing with microglia and inducing IFN-γ-associated neuroinflammatory pathways (73). Recent work has further demonstrated that CD8^+^ T cells exacerbate AD-like symptoms in mouse models of amyloidosis, extending the pathogenic relevance of these lymphocytes beyond tauopathy to amyloid-driven pathology (74). Collectively, these findings support a microglia-gated pathogenic axis in which CD8^+^ T cells may function as inflammatory amplifiers. However, direct evidence that this crosstalk culminates in synaptic elimination in human AD remains limited.

A distinct neurotoxic program has been identified among brain-infiltrating CD8^+^ T cells. Terrabuio et al. (2025) reported that CD103-negative CD8^+^ T cells promote neurotoxic inflammation in AD through granzyme K (GZMK)–protease-activated receptor 1 (PAR-1) signaling, independently of traditional perforin-dependent cytotoxicity (75). In parallel, GZMK expressed by subsets of CD8^+^ T cells and innate-like lymphocytes has been shown to act as an initiator protease that independently activates the entire complement cascade through direct membrane binding and cleavage of C4 and C2 (76). These observations delineate a non-canonical, lymphocyte-driven inflammatory program that operates in parallel with classical perforin-dependent cytotoxicity. The immunoproteasome, a specialized proteolytic complex induced in lymphocytes during inflammation, has also been shown to disturb neuronal metabolism and drive neurodegeneration in multiple sclerosis, raising the possibility that similar metabolic disruption may occur in AD when CD8^+^ T cells engage neuronal or glial targets (77).

Several mechanistic pathways may link pathogenic CD8^+^ T-cell programs to synaptic dysfunction. One putative model, extrapolated from neuroinflammatory and viral encephalitis systems, is direct cytotoxicity. In this model, CD8^+^ T cells recognize neuronal MHC-I-presented antigens and, in some paradigms, preferentially engage neuritic compartments. Such interactions can induce perforin-dependent electrical silencing in non-viral neuroinflammatory settings and MHC-I-restricted neuronal injury or apoptosis in viral encephalitis. However, whether analogous antigen-specific, MHC-I-restricted synaptic or neuritic attack occurs in human AD remains unknown (59, 63, 78). In AD, Aβ destabilizes the neuronal MHC-I/β2m complex, impairing its interaction with neural cell adhesion molecule 1 and disrupting synaptic signaling (79). Neuronal ApoE has also been linked to cell-autonomous regulation of MHC-I expression and selective neuronal vulnerability in the aged brain, although whether this pathway licenses CD8^+^ T-cell targeting in AD remains hypothetical (48). Although infiltrating CD8^+^ T cells show clonal expansion and antigen experience, and some TCRs have inferred viral specificities, the neuronal target antigen or antigens recognized by these cells have not been defined (9). Thus, direct antigen-specific synaptic killing by CD8^+^ T cells remains an important but unproven mechanism in human AD.

A second, more empirically supported model emphasizes indirect modulation through microglial reprogramming. In a 3D human neuroimmune axis model, infiltrating CD8^+^ T cells exacerbate neuronal and neuritic damage and amplify neuroinflammation through microglial activation (73). In P301S tauopathy mice, IFN-γ-producing T cells establish functional crosstalk with microglia (51), and hippocampal T-cell infiltration promotes neuroinflammation and cognitive decline in tau transgenic mice (72). These studies suggest that CD8^+^ T cells may act upstream of microglial dysfunction. However, they do not establish CD8^+^ T cells as direct executioners of synapse elimination. Synaptic loss may instead reflect indirect inflammatory amplification, altered glial phagocytic thresholds, or secondary neuronal stress. IFN-γ released by activated CD8^+^ T cells can drive persistent epigenetic shifts in neurons and synaptopathy, as demonstrated in encephalitis models (80). This suggests that cytokine-mediated neuronal reprogramming may represent an additional indirect pathway to synaptic dysfunction.

Consistent with indirect synaptic suppression, Unger et al. (2020) reported that anti-CD8 antibody-mediated depletion in APP/PS1 mice upregulated neuronal immediate early genes, including activity-regulated cytoskeleton-associated protein and neuronal PAS domain protein 4, and enriched pathways associated with calcium signaling, long-term potentiation, and glutamatergic synapses (12). This finding was interpreted as evidence that CD8^+^ T cells suppress synaptic plasticity in AD. At the same time, it suggests that brain-infiltrating CD8^+^ T cells are positioned to dynamically modulate synaptic function and that their removal releases a transcriptional brake on postsynaptic plasticity programs.

A third emerging pathway involves non-canonical complement activation. GZMK expressed by CD8^+^ T cells and innate-like lymphocytes can activate the complement cascade through direct cleavage of C4 and C2 (76), raising the possibility that T-cell-derived GZMK could amplify synaptic vulnerability via complement-mediated opsonization. However, direct evidence that GZMK triggers complement deposition at AD synapses is currently lacking. Therefore, the GZMK-complement-synapse axis in the context of pathogenic CD8^+^ T-cell programs should be viewed as a plausible but still unvalidated mechanism. **Figure 1** schematically depicts the principal pathogenic pathways through which brain-infiltrating CD8^+^ T cells may compromise synaptic integrity in AD.

**Figure 1:**
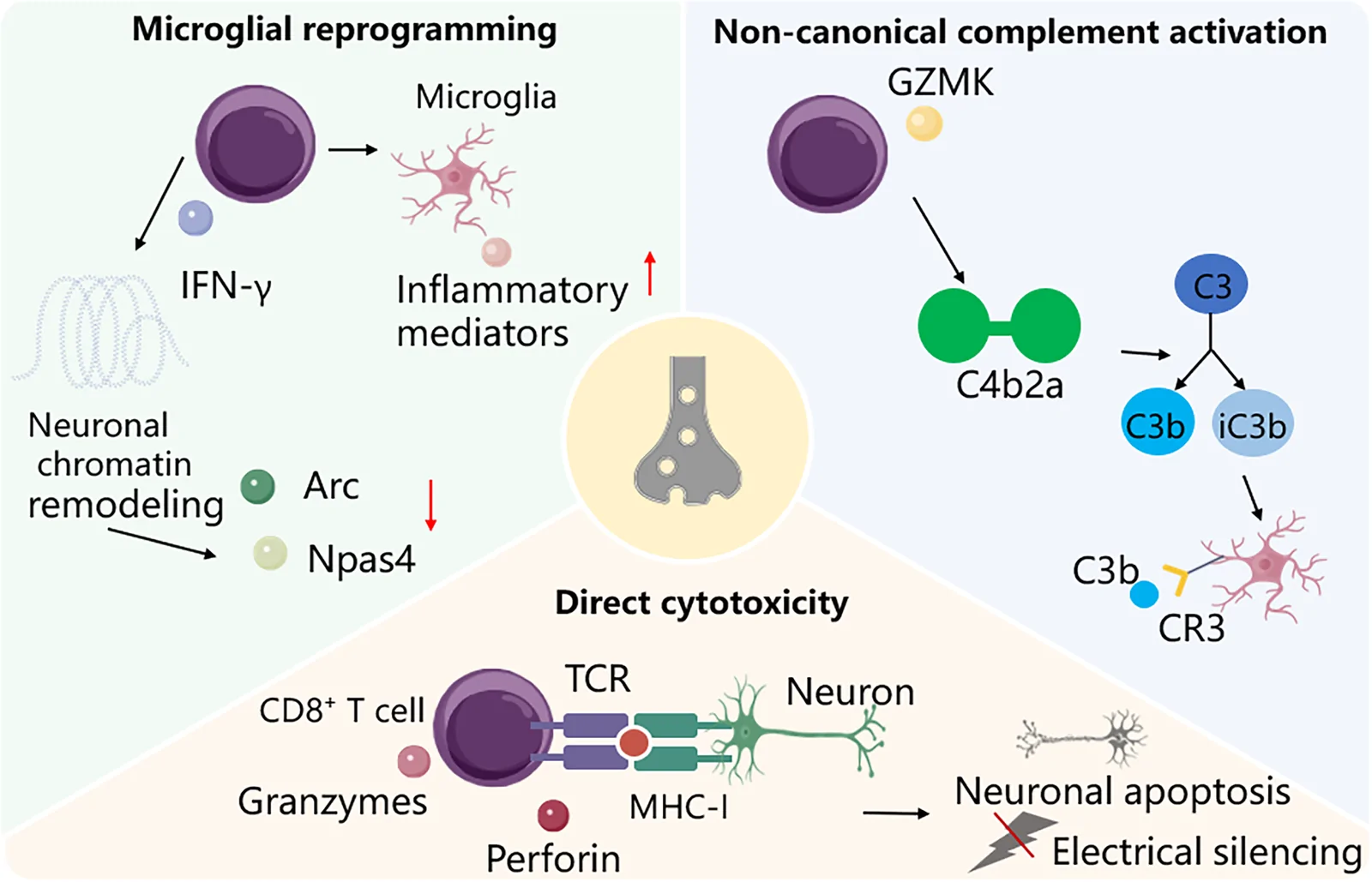
Proposed pathogenic CD8^+^ T cell–synapse axis in Alzheimer’s disease. (1) Direct cytotoxicity: TCR recognition of neuronal MHC-I triggers perforin/granzyme B, causing neuronal electrical silencing or apoptosis. (2) Microglial reprogramming: IFN-γ activates microglia to release inflammatory mediators and induce chromatin remodeling. (3) Non-canonical complement activation: GZMK cleaves C4 and C2, forming C4b2a convertase, which cleaves C3 to deposit C3b on synapses, promoting CR3-mediated phagocytosis.

Tissue-resident CD8^+^ T cells have also been shown to drive compartmentalized and chronic autoimmune damage against CNS neurons in non-AD contexts, reinforcing the principle that residency programming does not inherently confer neuroprotection (81). These observations delineate the pathogenic potential of brain-infiltrating CD8^+^ T cells, while protective programs have also been described in recent studies.

### 3.3 Protective and regulatory CD8^+^ T-cell programs and synaptic preservation

Although CD8^+^ T cells can promote neuroinflammation and synaptic dysfunction, accumulating evidence suggests that they may also exert context-dependent protective functions in AD. This protective capacity is particularly evident in loss-of-function studies. Su et al. (2023) identified a CXCR6^+^PD-1^+^ CD8^+^ T-cell population resident in the 5xFAD brain that limits amyloid accumulation and cognitive decline; ablation of these cells or genetic deletion of Cxcr6 worsened pathology, increased plaque burden, and exacerbated proinflammatory microglial activation (14). Similarly, Mason et al. (2025) reported that GZMK^+^ CD8^+^ T cells lacking classical cytotoxic molecules such as perforin, granzyme B, IFN-γ, and TNF exert neuroprotective effects in tauopathy by targeting distressed antigen-presenting microglia and restraining phosphorylated tau spread. In that model, deletion of CD8^+^ T cells accelerated neurodegeneration, promoted the emergence of microglia containing neuronal transcripts, and enhanced phosphorylated tau propagation (82). These observations provide strong evidence that CD8^+^ T cells are not uniformly pathogenic in AD and that selected CD8^+^ T-cell subsets may actively constrain neurodegenerative processes.

The CXCL16/CXCR6 chemokine axis appears to serve as a critical recruitment and retention signal for protective CD8^+^ T-cell populations in the AD brain (14). In this setting, microglial CXCL16 recruits or maintains CXCR6^+^PD-1^+^ CD8^+^ T cells, which in turn attenuate microglial proinflammatory cytokine production and restrain Aβ plaque accumulation. Similarly, GZMK^+^ CD8^+^ T cells in tauopathy appear to target distressed antigen-presenting microglia and limit phosphorylated tau propagation (82). These findings support a model in which protective CD8^+^ T cells act primarily as sentinels that gate microglial-mediated synaptic damage, shifting the glial niche from a neurotoxic state toward a more homeostatic or damage-containing state. In this framework, synaptic preservation is likely mediated less by direct neuronal engagement and more by restraint of maladaptive microglial activation, phagocytosis, and inflammatory amplification.

Importantly, GZMK expression should not be interpreted as uniformly pathogenic. Its functional consequence appears to depend on the cellular source, target cell, disease model, and inflammatory context. On one hand, CD103-negative GZMK^+^ CD8^+^ T cells can promote neurotoxic inflammation through PAR-1 signaling (75), and GZMK can activate the complement cascade through cleavage of C4 and C2 (76). On the other hand, GZMK^+^ CD8^+^ T cells lacking classical cytotoxic molecules can exert neuroprotective effects by targeting distressed antigen-presenting microglia and restraining tau propagation (82). These apparently opposing findings suggest that GZMK^+^ CD8^+^ T cells may not constitute a single functional entity. Rather, they may represent a family of context-dependent states whose effects are determined by co-expressed cytotoxic molecules, tissue niche, antigenic stimulation, and the activation state of neighboring microglia. In the protective context, GZMK^+^ CD8^+^ T cells may modulate complement-dependent synaptic pruning in a regulatory manner—potentially normalizing microglial phagocytic states or redirecting immune clearance toward damaged structures rather than viable synapses. However, direct evidence that protective GZMK^+^ CD8^+^ T cells regulate complement deposition at AD synapses is still lacking.

Beyond AD, analogous regulatory CD8^+^ T-cell subsets have been characterized in other neurological insults. Cai et al. (2022) identified a population of CD8^+^CD122^+^ T regulatory-like cells that infiltrate the ischemic brain via the CXCR3/CXCL10 axis and are reprogrammed to upregulate leukemia inhibitory factor receptor, transforming growth factor-alpha (TGF-α), and interleukin-10 (IL-10) (83). In that model, IL-10 dampened neuroinflammation, whereas epidermal growth factor-like transforming growth factor provided direct neuroprotection against ischemic injury. Earlier work by Bodhankar et al. (2015) demonstrated that IL-10-secreting B-cell therapy expanded CD8^+^CD122^+^ regulatory T cells within the CNS, correlating with reduced infarct volume and improved functional recovery (84). Although these studies were conducted in stroke models, they raise the possibility that analogous regulatory CD8^+^ T-cell subsets in the AD brain could secrete neurotrophic or anti-inflammatory factors that support synaptic resilience. Neuronal sensing of IL-12 has also been shown to induce neuroprotective CNS tissue adaptation and attenuate neuroinflammation, suggesting that cytokine-mediated neuroprotection may be a broader principle relevant to CD8^+^ T-cell-neuron interactions in AD (85). In addition, cellular rejuvenation strategies that protect neurons from inflammation-mediated cell death have recently been demonstrated, offering a translational perspective on how enhancement of endogenous protective mechanisms might complement immunomodulatory approaches (86).

An additional consideration is that the physiological contribution of CD8^+^ T cells to hippocampal neurogenesis and synaptic plasticity, documented in non-pathological settings, may persist during early AD stages before being overwhelmed by chronic inflammation (87). If so, therapeutic interventions that indiscriminately suppress CD8^+^ T cells could inadvertently eliminate not only pathogenic effectors but also resident or regulatory populations that support synaptic resilience. This underscores the necessity of subset-specific rather than pan-T-cell approaches in immunomodulatory strategies targeting the AD brain. **Figure 2** schematically summarizes the proposed protective CD8^+^ T cell–synapse axis.

**Figure 2:**
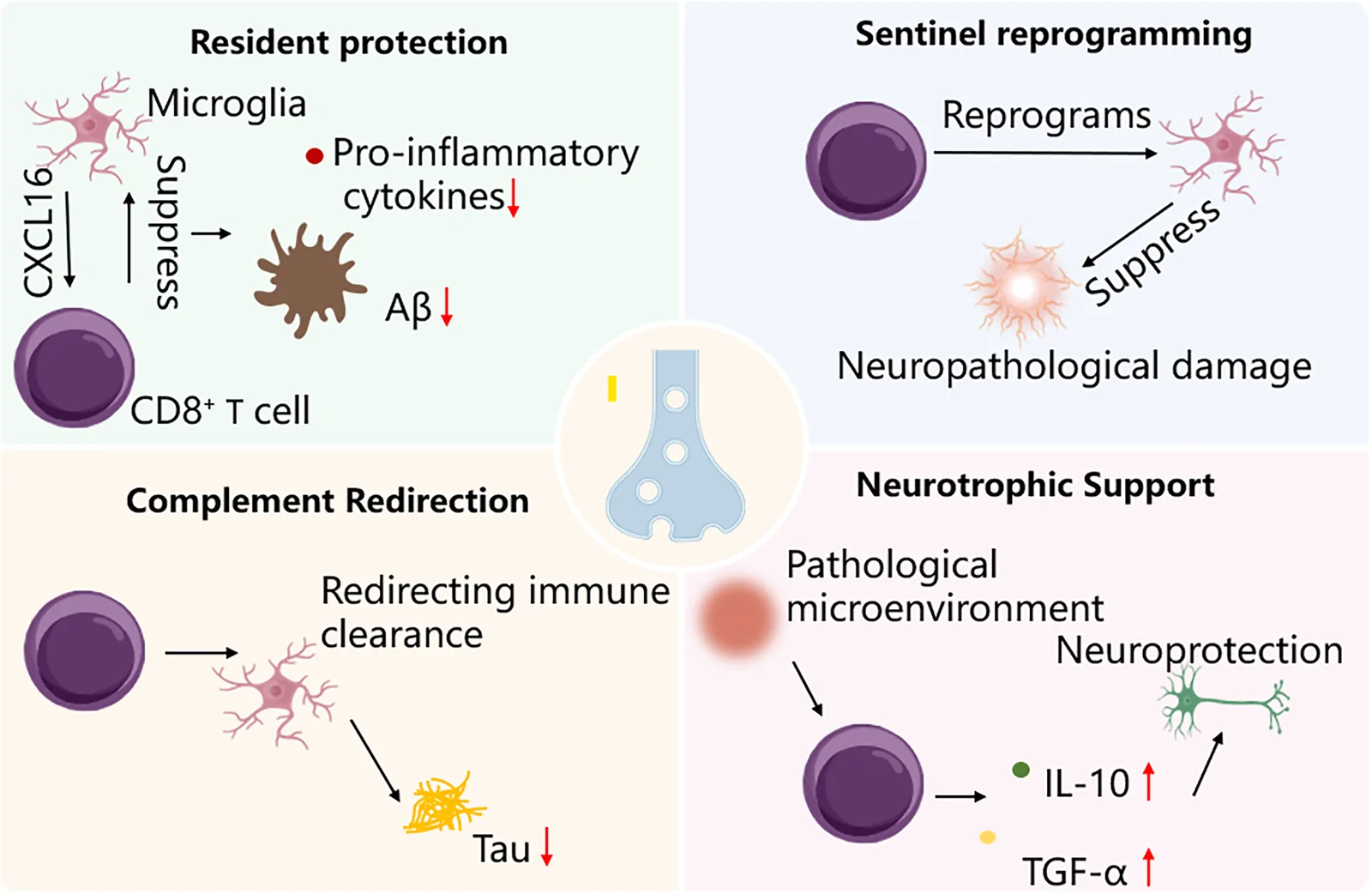
Proposed protective CD8^+^ T cell–synapse axis in Alzheimer’s disease. (1) CXCR6 axis: microglial CXCL16 recruits T cells to reduce Aβ and inflammation. (2) Sentinel reprogramming: T cells re-educate microglia to a homeostatic state, preserving synapses. (3) Complement redirection: GZMK modulates complement, redirecting microglial clearance toward damage rather than synapses, limiting tau spread. (4) Neurotrophic support: T cells, induced by the microenvironment, secrete IL-10 and TGF-α to suppress neuroinflammation and directly protect neurons.

### 3.4 Functional duality and contextual determinants at the synapse

The evidence reviewed above reveals a fundamental principle: the same CD8^+^ T-cell subset, molecular pathway, or homing receptor can produce diametrically opposed effects on synaptic integrity depending on context. GZMK^+^ CD8^+^ T cells drive neurotoxic inflammation via PAR-1 signaling in one AD model (75), yet slow tau propagation by eliminating distressed antigen-presenting microglia in another (82). CXCR6^+^ tissue-resident CD8^+^ T cells limit amyloid pathology in 5xFAD mice (14), but sustain microglia-mediated synapse elimination after West Nile virus infection (88). These divergent outcomes are not necessarily contradictory. Rather, they illustrate that the net effect of CD8^+^ T cells on synapses is shaped by multiple interdependent variables.

First, disease stage appears to be a critical switching factor. Early brain-infiltrating CD8^+^ T cells in AD models exhibit effector-like and pathogenic programs, whereas later populations acquire tissue-resident or exhaustion-like features with corresponding functional shifts (13). Second, the antigenic milieu determines whether CD8^+^ T cells engage in beneficial immune surveillance, harmful antigen-specific cytotoxicity, or bystander inflammatory activation. In the absence of defined cognate antigens in AD, clonal expansion may reflect viral reactivation, neoantigen exposure, autoimmune priming, or other forms of chronic antigenic stimulation, each with different implications for synaptic outcomes (9). Third, the functional state of engaged microglia acts as a gatekeeper. Protective CD8^+^ T cells can reprogram microglia toward homeostatic or damage-containing states (14), whereas pathogenic subsets may drive microglia toward hyperphagocytic and proinflammatory phenotypes that engulf viable synapses (51, 73). Fourth, anatomical niche matters. Meningeal, perivascular, and parenchymal CD8^+^ T cells encounter distinct cytokine environments, antigen-presenting cell populations, vascular interfaces, and complement landscapes, all of which may bias their functional output.

This contextual plasticity has profound implications for therapeutic strategy. Current evidence supports the presence, phenotypic heterogeneity, and disease association of CD8^+^ T cells in AD, but their causal role in synaptic degeneration remains inferential in humans and predominantly indirect in experimental models. Broad CD8^+^ T-cell depletion risks ablating protective tissue-resident populations alongside pathogenic effectors (14, 82), whereas indiscriminate immunosuppression may obscure stage-specific beneficial activities. The central challenge is therefore to define the combinatorial rules that determine whether a given CD8^+^ T-cell subset is more likely to preserve synaptic homeostasis or contribute to synaptic vulnerability in a particular patient at a particular disease stage. An at-a-glance summary of evidence strength is presented in **Table 1**, while **Supplementary Table 1** provides the corresponding detailed mechanistic narratives and translational constraints across human and rodent systems.

**Table 1:** Current evidence linking CD8^+^ T cells to synaptic regulation in AD.

Concept	Human observational studies	Animal models	*In vitro*/*ex vivo* studies	Core points
Presence and distribution of CD8^+^ T cells in AD	Moderate	Moderate	Limited	Detected in AD CSF and brain parenchyma; direct synaptic role unproven.
Reverse causality and translational constraints	Weak	Weak	Limited	Temporal causality unresolved; may reflect secondary recruitment to pre-existing pathology.
Phenotypic heterogeneity and context-dependent functional switching	Moderate	Moderate	Limited	Stage-dependent transitions between effector-like, resident, and exhaustion-like states.
Pathogenic CD8^+^ T-cell programs and synaptic damage	Moderate	Strong	Moderate	Pathogenic mechanisms supported in models; human synapse-level validation lacking.

## 4 Unresolved questions in the CD8^+^ T cell–synapse axis

The hypothesis that brain-infiltrating CD8^+^ T cells regulate synaptic integrity in AD remains compelling, but it is not yet an established mechanism, and several key questions continue to limit causal inference.

First, the relevant antigenic targets remain undefined on both sides of the axis. Although clonally expanded, antigen-experienced CD8^+^ T cells are consistently detected in the AD brain and CSF, and some TCRs show inferred viral specificities, the neuronal or synaptic antigens that might drive these responses have not been identified (9). Without defined cognate antigens, the model of MHC-I-restricted direct cytotoxicity cannot be formally tested in human AD tissue, and claims of antigen-specific synaptic attack remain extrapolated from viral encephalitis and demyelinating disorders (59, 63, 78). The same gap constrains the protective arm: the CXCR6^+^PD-1^+^ resident population that limits amyloid pathology and the GZMK^+^ CD8^+^ T cells that target distressed microglia in tauopathy models are presumed to be antigen-specific, yet their cognate antigens are equally unknown (14, 82). Until the antigenic specificities of both harmful and protective clones are resolved, antigen-directed interventions, whether tolerization of pathogenic clones or selective expansion of protective ones, remain out of reach.

Second, temporal and spatial causality remains unresolved for damage and protection alike. It is still unclear whether CD8^+^ T-cell infiltration precedes and initiates synaptic elimination, or instead amplifies synapse loss already triggered by amyloid, tau, complement activation, or other inflammatory insults. Human evidence is derived predominantly from end-stage post-mortem tissue and short-term *in vitro* models, making it difficult to resolve temporal order (12, 73). In addition, stage-dependent transitions from cytotoxic effector states to tissue-adapted or exhaustion-like phenotypes may bias post-mortem sampling toward functionally remodeled populations and obscure pathogenic activity at earlier stages of disease (13). A parallel temporal question concerns the protective subsets: it is unknown whether CXCR6-dependent residency and microglia-targeting surveillance constitute an early safeguard that is progressively lost as pathology advances, or instead a late compensatory response to established damage (14, 82). Longitudinal tracking of CSF TCR clonality across the MCI-to-dementia continuum, combined with *in vivo* measures of synaptic density such as SV2A PET (94), may help resolve the sequence of these opposing activities. Spatially, the field still lacks definitive evidence that infiltrating CD8^+^ T cells physically localize to vulnerable synaptic territories. Single-nucleus RNA-seq datasets report abundance and transcriptional state rather than direct colocalization (92), while spatially resolved transcriptomic and histological studies in AD and the aging brain reveal regionally heterogeneous immune and glial organization without yet resolving T-cell positioning relative to plaque-associated microglia, complement deposition, or synapse-loss hotspots (95–98). Spatial multi-omics approaches that capture TCR identity, immune effectors, complement components, and synaptic markers within the same tissue context will be essential.

Third, the operative mechanisms at human synapses remain unsettled in both directions. Indirect microglial reprogramming through IFN-γ currently has stronger experimental support, yet this framework places CD8^+^ T cells as upstream modulators rather than definitive executors of synapse elimination (51, 73). The recently described GZMK–complement axis further raises the possibility that T cells may promote synaptic opsonization through non-canonical complement activation, although whether GZMK activity can overcome local TREM2-dependent restraint of C1q signaling at AD synapses remains unknown (37, 76). For synaptic protection, the mechanisms are even less defined. Current models invoke indirect governance of microglial states and, by analogy with cerebral ischemia, the secretion of anti-inflammatory or neurotrophic factors by regulatory-like subsets (14, 82–84). However, none of these pathways has been directly linked to synapse-level readouts in AD tissue. Moreover, the physiological contribution of CD8^+^ T cells to hippocampal neurogenesis and synaptic plasticity raises an additional complication (87). Some protective functions may operate independently of pathology altogether, which would further complicate the interpretation of depletion experiments.

Fourth, the contextual determinants that switch CD8^+^ T cells between synaptotoxic and synaptoprotective outputs are unknown. The same effector molecules and homing pathways can produce opposite outcomes. For instance, GZMK^+^ CD8^+^ T cells drive neurotoxic inflammation through PAR-1 signaling in one AD model (75), yet slow tau propagation by eliminating distressed microglia in another (82). Similarly, CXCR6^+^ tissue-resident CD8^+^ T cells limit amyloid pathology in 5xFAD mice (14), but sustain microglia-mediated synapse elimination after West Nile virus infection (88). Disease stage, antigenic milieu, costimulatory context, and the functional state of the engaged microglia have all been proposed as switching variables (13, 88), but no study has systematically mapped how these factors determine the net effect of CD8^+^ T cells on synaptic integrity. Defining this balance is not merely academic, because it dictates whether therapeutic manipulation should aim to suppress, preserve, or selectively expand specific subsets.

Finally, translational validation remains limited for both complement-related and CD8^+^ T cell-related mechanisms of synaptic regulation. Therapeutic manipulation of T-cell checkpoints or trafficking pathways remains challenging to interpret in the absence of direct synaptic readouts, carries substantial immunological risk (99, 100), and must contend with the context-dependent duality of PD-1/PD-L1 signaling (69, 93). To consolidate these dispersed comparisons, **Table 2** juxtaposes rodent and human evidence across the key dimensions that currently limit translational inference.

**Table 2:** Key translational gaps between rodent models and human AD.

Dimension	Rodent evidence	Human evidence	Translational constraint	Refs.
Immune background	SPF mice have limited antigen-experienced memory relative to humans; some transgenic models show accelerated or altered immune aging features	Clonally expanded CD8^+^ T cells have been detected in CSF and, in some studies, brain tissue	Rodent immune ontogeny only partially recapitulates lifelong antigen exposure and age-associated immune remodeling in humans	(90, 91)
Neuronal MHC-I	Basal and inducible neuronal MHC-I expression has been reported in rodents, supporting the plausibility of direct cytotoxicity models	Evidence for neuronal MHC-I expression in the adult human cortex is limited and inconsistent, with reports strongest in selected neuronal populations	Direct neuronal antigen presentation in human AD remains unvalidated	(15, 45, 79)
T-cell detection	T-cell accumulation in parenchymal, perivascular, or meningeal compartments has been reported in multiple models, with stage/model variability	Human datasets show variable T-cell signals across methods; post-mortem studies are largely cross-sectional	The temporal relationship between T-cell entry and synaptic loss in human AD remains unresolved	(10, 12, 92)
Synaptic endpoints	In selected models, CD8-targeting interventions rescue synaptic molecular and structural readouts	Human immunomodulatory studies have not systematically incorporated synapse-specific biomarkers such as SV2A PET	Synaptic effects that appear causal in rodents remain inferential in humans	(12, 94, 101)
Functional duality	Stage- and context-dependent pathogenic and potentially protective T-cell states have been described in rodents	Phenotypic heterogeneity is suggested in human studies, but protective mechanisms remain poorly characterized	Context-dependent shifts between synaptotoxic and synaptoprotective states remain unresolved	(13, 14, 66)

## 5 Therapeutic implications and clinical outlook

### 5.1 CD8^+^ T cell immunomodulation: from depletion to subpopulation reprogramming

The functional duality of CD8^+^ T cells in AD—encompassing pathogenic effectors, CXCR6^+^PD-1^+^ tissue-resident memory cells with homeostatic potential (14), and functionally remodeled or exhaustion-like late-stage populations (13)—raises concerns about the clinical viability of broad depletion strategies. While preclinical models suggest that indiscriminate ablation may eliminate protective subsets alongside pathogenic ones, these observations await validation in human trials. Recent single-cell profiling in human tissue has identified clonal CD8^+^ T-cell populations in the leptomeninges of degenerative brain diseases, underscoring the need to dissect distinct functional roles rather than treating CD8^+^ T cells as a uniform cytotoxic population (89). In parallel, single-cell transcriptomic studies in AD models have identified CD103^-^CD8^+^ T cells as a distinct neurotoxic subpopulation that promotes inflammation via GZMK–PAR-1 signaling, independently of traditional perforin-dependent cytotoxicity (75). Conversely, IL-6 signaling has been genetically implicated in protective CD8^+^ T-cell–related phenotypes (102); pharmacological strategies targeting IL-6 trans-signaling may theoretically reduce neuroinflammation while preserving microglial homeostatic functions (103, 104). These findings collectively indicate that future immunomodulatory trials must move beyond binary “pro-inflammatory versus anti-inflammatory” frameworks and instead tailor interventions to distinct T-cell subpopulations. Selective blockade of GZMK–PAR-1 signaling could specifically disarm the CD103^-^ neurotoxic subset without compromising conventional perforin-dependent cytotoxicity or antiviral surveillance. Such a dual approach—selectively neutralizing pathogenic subsets while fostering protective T-cell functions—offers a more precise and potentially clinically viable paradigm than broad T-cell depletion. Realizing this dual approach, however, faces a practical bottleneck: as with other brain immune populations, current flow cytometry panels on post-mortem tissue remain poorly standardized and susceptible to enzymatic degradation of surface markers (105, 106).

T-cell–directed immunomodulation is now entering early clinical evaluation. In a recent first-in-human, phase 1b, randomized, double-blind trial (NCT05551741), IBC-Ab002—an Fc-engineered, short-lived anti-PD-L1 monoclonal antibody designed for transient, intermittent checkpoint blockade—demonstrated acceptable safety and tolerability in 40 participants with early AD across five ascending dose cohorts, with confirmed peripheral target engagement and transient CD4^+^ T-cell activation following each dose (107). Although efficacy data remain preliminary, this milestone suggests that T-cell immunomodulation is transitioning from preclinical speculation toward clinical evaluation, albeit a transition that demands exquisite subset specificity to avoid the autoimmune encephalitis risks documented with systemic checkpoint inhibitors (99, 100). It is important to emphasize that these therapeutic considerations assume CD8^+^ T cells contribute causally to synaptic pathology; should future studies confirm that their infiltration is purely secondary to neurodegeneration, the rationale for T-cell-directed immunomodulation would require reassessment.

### 5.2 ApoE4 stratification: the imperative for genotype-directed immunotherapy

ApoE4 status fundamentally reshapes both immune endophenotype and therapeutic risk, challenging the rationale for genotype-blind immunomodulation. Single-cell profiling reveals that ApoE4 carriers exhibit contraction of central memory CD8^+^ T cells alongside expansion of effector memory subsets, with 108 differentially expressed genes in the central memory compartment implicated in cell activation, cytotoxicity, and immunoregulatory interactions (108). Concurrently, this genotype perturbs innate immune compartments: ApoE4 enhances microglial antigen presentation and lipid-raft MHC-II clustering, creating a permissive environment for T-cell–microglia crosstalk that APOE3 brains may lack (109). Clinically, the significance of ApoE4 genotyping has been underscored by the anti-amyloid monoclonal antibody era: ApoE4 carriers, particularly homozygotes, experience substantially higher rates of amyloid-related imaging abnormalities (ARIA) following lecanemab or donanemab treatment, prompting regulatory agencies in Europe to restrict or carefully titrate these therapies in ϵ4/ϵ4 individuals (110). Whether ApoE4-associated ARIA risk extends to T-cell-directed immunomodulation remains to be determined, although the convergence of heightened immune reactivity and compromised vascular integrity in ApoE4 brains suggests that these patients may face amplified risks under systemic immunomodulation, whether complement-directed or T-cell-directed (111, 112). Consequently, AD immunotherapy may ultimately require ApoE4 stratification, as the risk–benefit profile of immune interventions appears fundamentally altered in ApoE4-driven pathophysiology.

### 5.3 Meningeal lymphatics: an adjunctive modulation axis with implications for CD8^+^ T cell antigen load

An alternative to direct immune cell targeting lies in restoring CNS waste clearance and immune surveillance through meningeal lymphatic modulation. Recent evidence demonstrates that glymphatic and meningeal lymphatic dysfunction drives Aβ and tau accumulation, neuroinflammation, and vascular impairment in AD, forming a vicious cycle that accelerates neurodegeneration (113). Preclinical systematic reviews indicate that interventions enhancing meningeal lymphatic drainage are beneficial. Examples include vascular endothelial growth factor C delivery and photobiomodulation. These interventions reduce amyloid burden, restore microglial homeostatic gene expression, and improve spatial memory in 5xFAD and APP/PS1 mice (114). These findings have spurred emerging clinical translation, including investigational approaches such as deep cervical lymphovenous anastomosis, for which efficacy data in AD remain preliminary (115). Notably, ApoE4 itself disrupts meningeal lymphatic function, increasing AD risk via Aβ clearance deficits (113). Sleep and arterial pulsatility critically regulate glymphatic efficiency, offering non-pharmacological therapeutic avenues that could be combined with immunomodulatory drugs to maximize clearance during the restorative sleep phase (113). By lowering parenchymal antigen burden, these clearance mechanisms may indirectly reduce the antigenic load driving CD8^+^ T-cell clonal expansion, offering an adjunctive strategy that could complement subset-specific immunomodulation without the infection risks associated with systemic immunosuppression. Furthermore, combining meningeal lymphatic enhancement with passive immunotherapy (e.g., anti-Aβ antibodies) has shown synergistic effects on Aβ clearance in preclinical models, suggesting that drainage restoration may serve as an adjuvant strategy within a combinatorial regimen rather than a standalone cure (115).

### 5.4 Biomarker-guided staging and multimodal trial design for CD8^+^ T cell–synapse interventions

The 2025 landscape of AD disease-targeted therapeutic trials reveals that inflammation-targeted interventions increasingly incorporate peripheral immune biomarkers, including T-cell surface markers and cytokines, as primary or secondary outcome measures (101). Synaptic biomarkers, particularly SV2A PET imaging with ¹¹C-UCB-J, now enable longitudinal quantification of synaptic density loss *in vivo* and permit monitoring of treatment effects on synaptic integrity (94). Postmortem quantitative immunohistochemistry further provides essential ground-truth data for interpreting these PET signals across neurodegenerative diseases (116, 117). High CSF TCR clonality, early SV2A PET decline, and ApoE4 positivity may define a CD8^+^ T-cell–dominant endophenotype responsive to localized immunomodulation or meningeal lymphatic enhancement, whereas robust complement tagging with minimal T-cell infiltration may indicate complement-directed therapy. This multimodal framework translates the CD8^+^ T-cell–synapse axis from mechanistic controversy into clinically actionable stratification. Adaptive immune modulation in AD demands precise calibration of disease stage, compartment, genetic background, and immune subset dynamics rather than a simple on–off switch.

## 6 Conclusion

This review has examined how synaptic regulation in AD may extend beyond a purely complement-centric framework to include an emerging CD8^+^ T cell–synapse axis. The complement system, while indispensable for developmental synaptic refinement and debris clearance, exhibits stage-dependent, region-dependent, and species-dependent roles in AD, such that aberrant synapse loss cannot be attributed to it exclusively. In parallel, brain-infiltrating CD8^+^ T cells appear to constitute a functionally heterogeneous population whose effects on synaptic homeostasis are likely shaped by disease stage, anatomical niche, antigenic milieu, and the functional state of engaged microglia.

Rather than uniformly promoting neurodegeneration or neuroprotection, these lymphocytes may drive synaptic elimination via multiple mechanisms: MHC-I-restricted cytotoxicity, IFN-γ-mediated microglial reprogramming, and non-canonical complement activation by GZMK. Conversely, they may preserve synaptic integrity through tissue-resident surveillance and homeostatic microglial governance. This functional duality renders simplistic therapeutic targeting untenable; broad depletion risks ablating protective subsets alongside pathogenic effectors, whereas indiscriminate immunosuppression may obscure stage-specific beneficial activities.

Future progress hinges on resolving whether infiltrating CD8^+^ T cells act as drivers or passengers of synaptic degeneration, defining the antigenic specificities of clonal CD8^+^ T cell expansions, establishing longitudinal spatiotemporal causality between T cell infiltration and synaptic loss, and defining the contextual determinants that switch these cells between synaptotoxic and synaptoprotective outputs. Translational efforts must move beyond binary inflammatory frameworks toward ApoE4-stratified, biomarker-guided, and subset-specific immunomodulation strategies that calibrate intervention to the dynamic neuroimmune landscape of each patient. Ultimately, precision immunomodulation in AD will likely require placing CD8^+^ T-cell biology within the broader complement-shaped neuroimmune landscape, while avoiding one-size-fits-all suppression of either pathway.
