# Supplementary material for: CD8+ t cell-mediated regulation of synaptic integrity in Alzheimer’s disease
Source: Front Immunol. 2026 Oct 1;17:1903001. doi: 10.3389/fimmu.2026.1903001 (PMC13669829; doi:10.3389/fimmu.2026.1903001)
Supplement: Supplementary file 1 [file Table1.docx]

# Supplementary Material

Supplementary Table 1. Current evidence linking CD8⁺ T cells to synaptic regulation in AD

| Concept | Human observational studies | Animal models | In vitro / ex vivo studies | Proposed mechanisms | Evidence summary | Refs. |
| --- | --- | --- | --- | --- | --- | --- |
| Presence and distribution of CD8⁺ T cells in AD | Clonally expanded CD8⁺ T-cell populations have been identified in AD-associated CNS compartments, including CSF and leptomeningeal niches | AD-like models show increased CD8⁺ T-cell recruitment/infiltration into the CNS | A 3D human neuroimmune axis model supports T-cell-associated neuritic injury, although direct synapse-specific validation remains limited | Recruitment to AD-associated CNS niches may reflect antigen-driven expansion, bystander inflammation, or secondary responses to ongoing neurodegeneration | CD8⁺ T cells are present in AD-associated CNS compartments, but their direct role in synaptic regulation remains unresolved | (9,89) |
| Reverse causality and translational constraints | Cross-sectional data cannot determine whether infiltration precedes or follows synaptic loss; snRNA-seq datasets have not uniformly detected CD8⁺ T-cell expansion in AD cortex/hippocampus | SPF mice lack antigen-experienced memory profiles typical of adult humans; premature immune aging in transgenic models complicates extrapolation | Limited | CD8⁺ T-cell accumulation may reflect secondary recruitment via chemokine release, BBB disruption, and antigen exposure rather than causal initiation of synaptic damage | The causal role of CD8⁺ T cells in human AD synaptopathy remains inferential; observed associations may reflect reverse causality or epiphenomenal responses | (12,16,17,90–92) |
| Phenotypic heterogeneity and context-dependent functional switching | Human data indicate that clonally expanded CD8⁺ T cells are functionally heterogeneous rather than uniformly cytotoxic | Experimental studies support stage-dependent transitions from effector-like pathogenic states to tissue-resident or exhaustion-like programs, with additional modulation by antigenic context and microglial state | Direct synaptic validation remains limited | Disease stage, antigenic milieu, and local microglial state may determine transitions between effector-like, tissue-resident, and exhaustion-like CD8⁺ T-cell states | CD8⁺ T-cell functions in AD are shaped by disease stage, antigenic milieu, and local neuroimmune context | (9,13,14) |
| Pathogenic CD8⁺ T-cell programs and synaptic damage | Clonally expanded, antigen-experienced CD8⁺ T cells are present in AD CSF and brain parenchyma; extravascular infiltrates correlate more robustly with tau than amyloid burden; direct evidence for antigen-specific, MHC-I-restricted synaptic attack remains absent. | CD8⁺ T cells infiltrate amyloid-laden regions and alter synapse-related gene expression; IFN-γ-producing T cells establish microglial crosstalk in P301S tau mice; CD103⁻ GZMK⁺ cells promote neurotoxic inflammation via PAR-1 signaling; GZMK activates complement by cleaving C4 and C2; anti-CD8 depletion upregulates neuronal IEGs (Arc, Npas4) and synaptic plasticity programs in APP/PS1 mice. | A 3D human neuroimmune axis model shows T cells exacerbate neuritic damage via microglial activation; MHC-I-restricted neuronal injury is established in viral encephalitis models; perforin-dependent electrical silencing is documented in non-viral neuroinflammatory paradigms. | (1) Direct cytotoxicity: TCR recognition of neuronal MHC-I triggers perforin/granzyme release, causing neuronal electrical silencing or apoptosis. (2) Microglial reprogramming: IFN-γ drives microglial activation and chromatin remodeling, indirectly lowering glial phagocytic thresholds and suppressing postsynaptic plasticity programs (Arc, Npas4, LTP, calcium signaling). (3) Non-canonical complement activation: GZMK expressed by CD8⁺ T cells cleaves C4 and C2 to generate C4b2a convertase, depositing C3b on synapses for CR3-mediated phagocytosis; GZMK also promotes neurotoxic inflammation via PAR-1 signaling independently of perforin-dependent cytotoxicity. | Multiple pathogenic routes are experimentally supported in AD models, but their relative contribution and direct synapse-level validation in human AD remain unresolved; antigen-specific, MHC-I-restricted synaptic attack and GZMK-driven complement deposition at synapses are mechanistically plausible yet unproven in human tissue. | (9,10,12,51,59,73,75,76) |
| Protective and regulatory CD8⁺ T-cell programs and synaptic preservation | Human data indicate clonal expansion and phenotypic diversity, but direct evidence for synaptoprotective mechanisms in AD remains limited. | CXCR6⁺PD-1⁺ tissue-resident CD8⁺ T cells limit amyloid accumulation and cognitive decline in 5xFAD mice; microglial CXCL16 recruits or maintains these cells. GZMK⁺ CD8⁺ T cells lacking classical cytotoxic molecules slow tau propagation by targeting distressed antigen-presenting microglia; CD8⁺ T-cell deletion accelerates neurodegeneration. CD8⁺CD122⁺ Treg-like cells infiltrate ischemic brain and secrete IL-10 and TGF-α. Physiological CD8⁺ T cells support hippocampal neurogenesis and synaptic plasticity. | Direct synapse-level validation of protective mechanisms in AD tissue remains lacking. | (1) CXCR6 axis: Microglial CXCL16 recruits CXCR6⁺PD-1⁺ CD8⁺ T cells, which attenuate proinflammatory microglial activation and restrain Aβ plaque accumulation. (2) Sentinel reprogramming: GZMK⁺ CD8⁺ T cells lacking perforin, granzyme B, IFN-γ, and TNF target distressed antigen-presenting microglia, shifting the glial niche toward a homeostatic state and limiting phospho-tau spread. (3) Complement redirection: GZMK⁺ CD8⁺ T cells modulate complement-dependent synaptic pruning in a regulatory manner, potentially normalizing microglial phagocytic thresholds and redirecting immune clearance toward damaged structures rather than viable synapses. (4) Neurotrophic support: CD8⁺CD122⁺ Treg-like cells secrete IL-10 and TGF-α to suppress neuroinflammation and directly protect neurons; basal CD8⁺ T-cell populations maintain hippocampal neurogenesis and synaptic plasticity. | Selected CD8⁺ T-cell subsets constrain neurodegeneration through microglial governance, complement modulation, and neurotrophic signaling; direct human synapse-level validation is lacking, and the functional consequence of GZMK expression depends on cellular source, target cell, and inflammatory context. | (14,76,82,83,87) |
| Context-dependent functional duality and neuroimmune modulation | Direct evidence remains limited; phenotypic diversity suggests identical markers cannot unambiguously classify cells as harmful or protective | GZMK⁺ CD8⁺ T cells drive neurotoxic inflammation via PAR-1 in one model yet slow tau propagation by eliminating distressed microglia in another; CXCR6⁺ cells limit amyloid in 5xFAD but sustain synapse elimination after West Nile virus infection | Limited | Disease stage, antigenic milieu, costimulatory context, and engaged microglial state determine functional output; microglial gating, complement-related pathways, and immune checkpoint signaling (PD-1/PD-L1, TIM-3) jointly modulate whether CD8⁺ T-cell responses become synaptotoxic or adaptive | The net effect on synapses is not subset-intrinsic but shaped by broader neuroimmune circuitry; therapeutic manipulation must account for this context-dependent duality rather than treating CD8⁺ T cells as uniformly cytotoxic | (14,69,70,75,76,82,88,93) |
